# Patterns and outcomes of individuals admitted at emergency units following intentional self-harm in Northern Uganda

**DOI:** 10.1007/s44192-024-00115-z

**Published:** 2024-11-25

**Authors:** Mark Mohan Kaggwa, Joan Abaatyo, Keneth Opiro, Margret Sikoti, Felix Bongomin

**Affiliations:** 1 https://ror.org/009z39p97 grid.416721.7 0000 0001 0742 7355 Forensic Psychiatry Program, St Joseph’s Healthcare Hamilton, 100 West 5th, Hamilton, ON L89 3K7 Canada; 2 grid.25073.33 0000 0004 1936 8227 Department of Psychiatry and Behavioral Sciences, McMaster University/St Joseph’s Healthcare Hamilton, 100 West 5th, Hamilton, ON L89 3K7 Canada; 3 Department of Psychiatry, School of Medicine, King Ceasor University, Kampala, Uganda; 4 https://ror.org/007pr2d48 grid.442658.9 0000 0004 4687 3018 Department of Psychiatry, Uganda Christian University, Kampala, Uganda; 5 https://ror.org/042vepq05 grid.442626.0 0000 0001 0750 0866 Faculty of Medicine, Gulu University, Gulu, Uganda; 6 https://ror.org/00ew8c753 grid.440165.2 0000 0004 0507 1799 . Mary’s Hospital Lacor, Gulu, Uganda; 7 https://ror.org/00tbh0a59 grid.459649.3 0000 0004 0500 5433 Department of Internal Medicine, Gulu Regional Referral Hospital, Gulu, Uganda

**Keywords:** Self-harm, Intentional self-harm, Uganda, Emergency care

## Abstract

We retrospectively reviewed charts of 253 self-harming patients admitted to emergency units in Northern Uganda in 2021. Twenty-two (8.7%) died by suicide, especially due to organophosphate poisoning (n = 14, 63.6%). Regarding self-harm management, observed differences were noted in the type of hospital and the use of antidotes between public and private facilities. There is a need for more studies and a multisector approach to prevent and treat self-harm in Uganda.

**Supplementary Information:**

The online version contains supplementary material available at 10.1007/s44192-024-00115-z.

## Short communication

Intentional self-harm is a global public health crisis that requires urgent attention. Every year, millions of people harm themselves intentionally, often with fatal consequences [1, 2]. Self-harm is one of the main reasons for emergency hospital admissions worldwide [3]. Many of these patients die at the emergency department (ED), while others survive but face a high risk of suicide or repeated self-harm [4]. These repeated attempts further increase the demand for the desired services and resources. Self-poisoning is a prevalent method of intentional self-harm worldwide, with varying incidence rates and patterns across regions [5, 6]. During the COVID-19 pandemic, studies from several African countries, including Egypt and Uganda, revealed alarming trends in suicide attempts and fatalities due to self-poisoning [7–9]. These rates were notably higher among young adults and were linked to socio-economic pressures, mental health issues, and interruptions in healthcare services [7, 8].

In Uganda, a low-income country with a fragile health system especially in post-conflict northern region, self-harm poses a serious challenge**.** The health sector, especially mental health, is underfunded and understaffed, making it difficult to provide adequate care for self-harm patients and prevent ED visits [10]. To worsen the situation, the speciality of emergency medicine is a juvenile profession, with most of its services relying on general practitioners. Hospital supplies, such as antidotes for many agents that are commonly used in self-harming, have chronically been inadequate in the country, potentially leading to many preventable deaths [11, 12]. For example, in 2020, over one in six self-harm related hospital visits in Southwestern Uganda resulted in death [9].

To understand the system's needs and future projections, studies on the burden of self-harm and its related factors provide insight into the situation. Various studies have shown prevalent levels of self-harm and suicide in many parts of Uganda, affecting various groups of people [9, 13–15]. However, there is a lack of research on this issue, especially in northern Uganda. A region grappling itself out of a long-standing war and unsettlement in the neighbouring countries, in the background of very high poverty levels [16–20]. Moreover, no studies have focused on the EDs that deal with the most severe cases of self-harm. Therefore, this study aimed to identify patterns and outcomes of individuals admitted for intentional self-harm in Northern Uganda, thus, providing insights into systemic needs and guiding effective interventions and resource allocation to reduce self-harm in this vulnerable population.

We conducted a prospective analysis to assess poisoning cases in two Northern Uganda’s emergency units [21]. For the present analysis, we included individuals admitted after episodes of intentional self-harm and excluded those admitted for accidental ingestion, non-self-inflicted harm, follow-up care, or non-emergency reasons to ensure the study focused on acute self-harm incidents.

In the analysis of the 253 patients, most were young males, treated at a private facility. Alcohol-related self-harming [i.e., deliberately use of alcohol as a method of self-harm] (44.7%) was the commonest method of self-harming, followed by organophosphate poisoning (31.2%), and medications/drugs overdose (9.9%). Almost two-thirds of the patients who self-harmed did not receive any antidotes, and 8.7% died (Table 1).

**Table 1:** Participant characteristics distribution across completed suicide

Variable	All participants (253) n (%)	Suicide		p-value
		Yes 22 (8.7)	No 231 (91.3)	
Age group				
< 21	38 (15.0)	1 (4.6)	37 (16.4)	0.226
21–40	131 (51.8)	10 (45.4)	121 (53.5)	
41–60	57 (22.5)	8 (36.4)	49 (22.5)	
> 60	20 (7.9)	3 (13.6)	17 (7.5)	
Not reported	7 (2.8)	0	7 (3.0)	
Sex				
Female	85 (33.6)	6 (27.3)	79 (34.3)	0.511
Male	168 (66.6)	16 (72.7)	152 (65.7)	
Type of poison				
Medication	25 (9.9)	0	25 (10.8)	0.005
Others	36 (14.2)	1 (4.5)	35 (15.1)	
Organophosphate	79 (31.2)	14 (63.6)	65 (28.1)	**0.005**
Alcohol	113 (44.7)	7 (31.8)	106 (45.9)	
Antidote given				
No	165 (65.2)	7 (31.8)	158 (68.4)	**0.001**
Yes	88 (34.8)	15 (68.2)	73 (31.6)	
Type of hospital attended				
Public	92 (36.4)	2 (9.1)	90 (39.0)	**0.005**
Private	161 (63.6)	20 (90.9)	141 (61.0)	
Median length of hospital stays (days) [median (IQR)]	1 (0–3)	1 (0.2)	1 (0.3)	0.838

*p-value < 0.05 = significant difference

When examining the types of poisons, there was a clear predominance of organophosphates and medication/drug use among females compared to males. In contrast, alcohol was significantly predominantly used by males than females (S1 Table 1).

Death was more likely among patients who used organophosphates, who were admitted to private facilities, and who received potential antidotes. A statistically significantly higher proportion of individuals received antidotes in the private facility than in the public facility (41.6% vs. 22.8%; *p* = 0.003). However, the private hospital admitted most of the patients with organophosphate and drug overdose, while the public facility was dominated by alcohol-related self-harm (S1 Table 2).

This study revealed the prevalence of suicide following intentional self-harm at 8.7% with clear predominance of organophosphate poisoning and medication/drug use among females. Death was higher among patients who used organophosphates, who were treated at private facilities, and received antidotes. Compared to private hospitals, alcohol-related self-harm was more common at the public facility.

The prevalence of suicide reported in this study was much higher than the national suicide prevalence rate in 2019 (4.6%) [22]. However, the rate was lower than 16.6% from selected health facilities in Southwestern Uganda during the first year of the COVID-19 pandemic, a period that was characterized by increased rates of suicide [9]. In contrast, the present study, the reported prevalence rates in Southwestern Uganda exclude self-harm cases from resource-rich tertiary hospitals that may potential access to antidotes to commonly used items for self-harming by many Ugandans, such as atropine, thus, potentially affecting overall prevalence rates [23].

The observed higher mortality secondary to the use of organophosphate is likely attributed to delays in reaching the hospital, lack of antidotes and resuscitation resources, and potentially overwhelming workload to the already few staff in the EDs. Ugandans have predominantly used organophosphates in self-harming due to easy access in an agriculture-dependent economy [9]. The high burden of this problem and the lack of resources in public-funded hospitals may be the driver to many individuals receiving services from private hospitals—which may have most of the needed resuscitation resources and antidotes. Uganda is among the countries with the highest alcohol-consuming countries in Africa, with the poor mainly engaging in excessive use [24]. Many crude forms of alcohol are available, and individuals struggling with various stressors may opt to “*drink down their problems*” [24]. The demographic of such individuals may not afford private services, thus their high numbers in public facilities.

To prevent these unnecessary deaths, Uganda needs to invest more in health care, especially mental health, and emergency care services**.** We recommend that EDs be supplied with adequate antidotes, that organophosphates and other dangerous drugs be regulated more strictly, and that mental health services be expanded for people who self-harm or have suicidal thoughts. We also suggest that efforts be made to reduce poverty and alcohol use, which are linked to self-harm and suicide. In conclusion, we call for a multisectoral approach to address self-harm as a growing public health issue in Uganda.

## Supplementary Information

Below is the link to the electronic supplementary material.

Supplementary file1 (DOCX 15 KB)

## Data Availability

Data will be provided upon request from the corresponding author.
