# Supplementary material for: Patterns and outcomes of individuals admitted at emergency units following intentional self-harm in Northern Uganda
Source: Discov Ment Health. 2024 Nov 25;4(1):59. doi: 10.1007/s44192-024-00115-z (PMC11589079; doi:10.1007/s44192-024-00115-z)
Supplement: Supplementary file 1 — Supplementary file1 (DOCX 15 KB) [file 44192_2024_115_MOESM1_ESM.docx]

**S1 Table 1: Distribution of type of poison across sex**

| **Variable** | **Sex** | | **p-value** |
| --- | --- | --- | --- |
|  | **Female**  85 (33.6) | **Male**  168 (66.6) |  |
| **Type of poison** | | | |
| Organophosphate | 39 (45.9) | 40 (23.8) | **<0.001** |
| Medication | 15 (17.7) | 10 (5.9) |  |
| Alcohol | 9 (10.6) | 104 (61.9) |  |
| Others | 22 (25.9) | 14 (8.3) |  |
| *p-value < 0.05 = significant difference | | | |

**S1 Table 2: Analysis as per health facility**

|  | **Type of hospital** | |  | **P-value** |
| --- | --- | --- | --- | --- |
|  |  | **Public**  92 (36.4) | **Private**  161 (63.6) |  |
| **Type of poison** | Organophosphate | 16 (17.4) | 63 (39.1) | **<0.001** |
|  | Medication | 5 (5.4) | 20 (12.4) |  |
|  | Alcohol | 71 (77.2) | 42 (26.1) |  |
|  | Others | 0 | 36 (22.4) |  |
| **Antidote given** | No | 71 (77.2) | 94 (58.4) | **0.003** |
|  | Yes | 21 (22.8) | 67 (41.6) |  |
